# Laparoscopy for the Treatment of Congenital Hernia: Use of Surgical Meshes and Mesenchymal Stem Cells in a Clinically Relevant Animal Model

**DOI:** 10.3389/fphar.2020.01332

**Published:** 2020-09-25

**Authors:** Federica Marinaro, Javier G. Casado, Rebeca Blázquez, Mauricio Veloso Brun, Ricardo Marcos, Marta Santos, Francisco Javier Duque, Esther López, Verónica Álvarez, Alejandra Usón, Francisco Miguel Sánchez-Margallo

**Affiliations:** ^1^ Stem Cell Therapy Unit, Jesús Usón Minimally Invasive Surgery Centre, Cáceres, Spain; ^2^ CIBER de Enfermedades Cardiovasculares, Madrid, Spain; ^3^ Department of Small Animal Clinics, Center of Rural Science, Federal University of Santa Maria (UFSM), Santa Maria, Brazil; ^4^ Laboratory of Histology and Embryology, Department of Microscopy, Abel Salazar Institute of Biomedical Sciences, University of Porto, Porto, Portugal; ^5^ Animal Medicine Department, Faculty of Veterinary Medicine, University of Extremadura, Cáceres, Spain; ^6^ Scientific Direction, Jesús Usón Minimally Invasive Surgery Centre, Cáceres, Spain

**Keywords:** mesh, congenital hernia, mesenchymal stem cells, animal model, hernia repair, stem cell therapy, abdominal hernia, laparoscopy

## Abstract

More than a century has passed since the first surgical mesh for hernia repair was developed, and, to date, this is still the most widely used method despite the great number of complications it poses. The purpose of this study was to combine stem cell therapy and laparoscopy for the treatment of congenital hernia in a swine animal model. Porcine bone marrow-derived mesenchymal stem cells (MSCs) were seeded on polypropylene surgical meshes using a fibrin sealant solution as a vehicle. Meshes with (cell group) or without (control group) MSCs were implanted through laparoscopy in Large White pigs with congenital abdominal hernia after the approximation of hernia borders (implantation day). A successive laparoscopic biopsy of the mesh and its surrounding tissues was performed a week after implantation, and surgical meshes were excised a month after implantation. Ultrasonography was used to measure hernia sizes. Flow cytometry, histological, and gene expression analyses of the biopsy and necropsy samples were performed. The fibrin sealant solution was easy to prepare and preserved the viability of MSCs in the surgical meshes. Ultrasonography demonstrated a significant reduction in hernia size 1 week after implantation in the cell group relative to that on the day of implantation (*p* < 0.05). Flow cytometry of the mesh-infiltrated cells showed a non-significant increase of M2 macrophages when the cell group was compared with the control group 1 week after implantation. A significant decrease in the gene expression of *VEGF* and a significant increase in *TNF* expression were determined in the cell group 1 month after implantation compared with gene expressions in the control group (*p* < 0.05). Here, we propose an easy and feasible method to combine stem cell therapy and minimally invasive surgical techniques for hernia repair. In this study, stem cell therapy did not show a great immunomodulatory or regenerative effect in overcoming hernia-related complications. However, our clinically relevant animal model with congenital hernia closely resembles the clinical human condition. Further studies should be focused on this valuable animal model to evaluate stem cell therapies in hernia surgery.

## Introduction

Internal organs can move from their normal position in the body and slip or protrude through weakened muscles and connective tissue, thereby resulting in hernias and pelvic organ prolapses (Baylón et al., 2017). The abdominal wall is particularly vulnerable to weaknesses, defects, or holes that may be due to iatrogenic causes, trauma, or congenital defects (Pulikkottil et al., 2015); these vulnerabilities may lead to the herniation of internal viscera. The physical location of the protrusion outward from the anterior abdominal wall is usually used to classify hernias according to type: inguinal hernias are protrusions outward of soft tissues through the inguinal canal; umbilical hernias occur in correspondence with the umbilicus; epigastric hernias are situated between the umbilicus and chest cavity; and incisional hernias occur through a previously made incision in the abdominal wall (Wales and Holloway, 2019). Many hernias are not accompanied by symptoms, except for the presence of a bulge in the abdomen. When a hernia opening in the abdominal wall is too narrow, it is defined as “incarcerated” and causes pain and obstruction of the intestines. Incarcerated hernias, in which the blood supply to hernia tissues is compromised, are defined as “strangulated” and can be associated with symptoms of pain, nausea, vomiting, peritonitis, septicemia, and circulatory failure (Kavic, 2005; Birindelli et al., 2017).

Repairing irregularities in the abdominal wall is necessary but challenging. For hernia repair, surgeons usually resort to an open suturing technique and/or mesh implantation through open surgery procedures or laparoscopic approaches. However, in the treatment of abdominal hernias, the application of surgical meshes has proved to be more effective than suturing (Finan et al., 2009). Surgical meshes are sterile, chemically and physically inert prosthetic materials that guarantee the reinforcement of the abdominal wall such that hernia recurrence is prevented when they are used (López-Cano et al., 2018). Nevertheless, implantation of a prosthetic material, despite its inertness, can lead to bacterial growth and infection that can delay wound healing. Concurrently, surgical meshes can trigger an exacerbated and chronic inflammatory reaction, leading to wound healing but also to foreign body reaction and the formation of scar tissues. A high proliferation of fibroblasts during the wound-healing phase has been linked to inflammation and fibrosis, which thereby cause contraction and shrinkage of the mesh (Baylón et al., 2017). Wound-healing-related issues, together with surgical complications, may result in paresthesia, pain, adhesions, fistulas, scar entrapment of nerves, infection, mesh migration, erosion, and rejection; these require consequent excision (Baylón et al., 2017; Klinge and Klosterhalfen, 2018). Researchers, physicians, and surgeons have been fighting a two-front war for many years, trying to improve surgical meshes and their applications. Regarding progress in surgical implantation, the laparoscopic approach for hernia repair was proposed as an alternative to traditional open surgery in the early 1990s (Eker et al., 2013). Although there remains a debate in defining whether open surgery or laparoscopy is the “gold standard” for hernia repair (Al Chalabi et al., 2015), surgeons have recently pointed toward the use of robot-assisted surgery (Carbonell et al., 2018). Additionally, advances in hernia surgery deal with primary defect closure, retrorectus mesh placement, and concomitant component separation (Vorst et al., 2015).

On the other hand, the improvement in surgical meshes has been focused on identifying and making use of the most appropriate material. More than 200 types of meshes were reported in 2013 (Klinge et al., 2013); these meshes have different mechanical properties, pore sizes, weight, density, constitution (monofilament or twisted), manufacturing processes (extrusion or knitting), anisotropy, and type of material (synthetic non-absorbable, mixed or composite, and biological) (Rastegarpour et al., 2016). Moreover, many biocompatible coatings have been developed to modify surgical mesh surfaces and are aimed at protecting the prosthesis from degradation, decreasing postsurgical inflammation, minimizing foreign body reaction, reducing the risk of infections, and decreasing adhesions (Majumder et al., 2015; Baylón et al., 2017; Bredikhin et al., 2020). Because of their huge therapeutic potential, stem cells have been one of the focuses of biomedical researchers in the last 20 years. Stem cells have a wide range of applications in many different diseases (Sánchez et al., 2012; Reisman and Adams, 2014; Rajabzadeh et al., 2019) and are now the targets of a multitude of clinical trials^1^ aimed at treating pathological conditions such as Crohn’s disease, urinary incontinence, multiple sclerosis, diabetes, rheumatoid arthritis, glioblastoma, and myocardial infarction. Stem cell therapy has also been applied to mesh-aided hernia repair to improve the healing outcome of damaged tissues. However, contradictory results have been obtained (Marinaro et al., 2019).

Previous studies from our group have demonstrated that mesenchymal stem cells (MSCs) reduce adverse inflammation following surgical mesh application in a murine incisional hernia model (Blázquez et al., 2018) by promoting macrophage polarization towards an anti-inflammatory and pro-regenerative M2 phenotype (Blázquez et al., 2016). In the present study, we have investigated a new approach for the treatment of abdominal hernias. Here, we propose the combined use of surgical meshes with MSCs for controlling an adverse inflammatory response following the implantation of mesh. In addition, this study was performed in a clinically relevant animal model (swine model of congenital abdominal hernia) by using minimally invasive procedures (laparoscopic approach). In this animal model, we have investigated: (i) the use of fibrin sealant as a vehicle to favor the adhesion of MSCs onto surgical meshes; (ii) the optimization and application of laparoscopic surgical procedures for the implantation of surgical meshes in a swine model of congenital abdominal hernia; and (iii) the evaluation of the effects of MSCs on mesh-repaired hernias. We demonstrated that fibrin sealants allow the adhesion of stem cells onto surgical meshes. Laparoscopy is a feasible approach for the successful implantation of stem cell-coated meshes. Our animal model with congenital hernia, which closely resembles the conditions of human patients with the same hernia, should be used for further preclinical studies. Although stem cell-based therapies have demonstrated a therapeutic potential in murine models (and under *in vitro* conditions), our experimental approach in this large animal model did not reveal any important contribution of stem cell therapy. It is important to note that further research is necessary to optimize the implantation of these cells in a real surgical context.

## Materials and Methods

### Ethical Considerations

The Ethics Committee on Animal Experiments of the Jesús Usón Minimally Invasive Surgery Centre (JUMISC), Cáceres, Spain, validated all the experimental procedures according to the recommendations outlined by the local government (Junta de Extremadura) and EU Directive 2010/63/EU of the European Parliament on the protection of animals used for scientific purposes. Housing, care, and husbandry of all the animals used throughout the study were carried out in the animal facility of the JUMISC.

### Isolation, Expansion, and Characterization of Allogeneic Porcine Bone Marrow-Derived Mesenchymal Stem Cells

A Large White pig (3 months old and 25 kg) was euthanized, and allogeneic bone marrow-derived MSCs (BM-MSCs) were obtained from its femurs by using a needle and syringe. BM-MSCs were isolated and characterized as previously described (Casado et al., 2012). Briefly, the mononuclear cells were collected from the cell suspension by filtration through a 40 µm nylon mesh (Fisher Scientific, Leicestershire, UK) and centrifugation in Histopaque-1077 solution (Sigma-Aldrich, St. Louis, MO). After washing with phosphate-buffered saline (PBS), the mononuclear cells were resuspended in complete cell culture medium, prepared with Dulbecco’s modified Eagle’s medium, 10% fetal bovine serum (FBS) (Sigma-Aldrich), 5 μl/ml amphotericin B (Fungizone), 1% glutamine, and 1% penicillin/streptomycin (Lonza, Basel, Switzerland), seeded into tissue culture flasks, and incubated at 37°C and 5% CO_2_. The non-adherent hematopoietic cells were removed after 48 h of incubation, whereas the adherent cells were passaged upon 80–90% confluence. The phenotypic characterization of BM-MSCs at passages 4–6 was performed by using a FACSCalibur™ Flow Cytometry System (BD Biosciences, CA, USA). Approximately 2 × 10^5^ cells were incubated for 30 min at 4°C with adequate concentrations of porcine fluorescein isothiocyanate-conjugated monoclonal antibodies against Integrin beta-1 (CD29), CD44 antigen (CD44), Thy-1 antigen (CD90), Endoglin (CD105), CD45 antigen (CD45), Swine leukocyte antigen class 1 (SLA-1), and Swine leukocyte antigen class 2 (SLA-2) (Bio-Rad, CA, USA), according to the manufacturer’s instructions. Isotype-matched negative control antibodies were used in the experiments. The CellQuest software (BD Biosciences, CA, USA) was used to analyze viable cells after the acquisition of 10^5^ events by using forward and side scatter characteristics. The mean fluorescence intensity (MFI) was determined relative to the MFI of its negative control to obtain the mean relative fluorescence intensity. As performed in our previous study (Casado et al., 2012), BM-MSCs were cultured for 21 days with differentiation medium (Gibco Life Sciences, Rockville, MD, USA) and stained with Oil Red O, Alcian Blue, and Alizarin Red S for the assessment of their potential toward adipogenic, chondrogenic, and osteogenic differentiation, respectively (Mok et al., 2008).

### Fibrin Sealant Admixture, Fibrin Clotting, and Cell Viability Assay of Mesenchymal Stem Cells

A fibrin sealant vehicle for allogeneic MSCs was prepared by using commercially available fibrin sealant Tisseel^®^ (Baxter, USA; product number 1504516). This product consists of two separated components: a “thrombin solution” (500 IU/ml thrombin) and a “sealer protein solution” (91 mg/ml fibrinogen and synthetic aprotinin). These solutions are mixed in a ratio of 1:1 to prepare a ready-to-use fibrin solution. To determine the optimal mixture for mesh coating, BM-MSCs were detached from flasks with 0.25% trypsin solution and counted. Around 5 × 10^4^ cells were resuspended in 0, 25, 50, 75, or 100 µl of complete cell culture medium and mixed with 100, 75, 50, 25, or 0 µl thrombin solution (pH 7.2, from Tisseel^®^; product number 1504516), respectively. Afterwards, these suspensions were mixed in a 1:1 ratio with the sealer protein solution (from Tisseel^®^; product number 1504516) (**Table 1**) and tested according to clotting capability and cell viability. Clotting capability was visually assessed, comparing clotted and gelatinous fibrin hydrogels against the unclotted liquid solutions for each mixture of thrombin solution, cell culture medium, and “sealer protein solution.”

**Table 1:** Composition of the culture medium (Dulbecco’s modified Eagle’s medium) and fibrin sealant mixtures for clotting capability assessment and cell viability assays.

Culture medium + fibrin sealant mixtures		
Complete cell culture medium (µl)	Thrombin solution (thrombin 500 UI/ml) (µl)	Sealer protein solution (fibrinogen 91 mg/ml) (µl)
0	100	100
25	75	100
50	50	100
75	25	100
100	0	100

The Cell Counting Kit-8 (CCK-8) assay (Merck KGaA, Darmstadt, Germany) was used according to the manufacturer’s instructions to determine cell viability. BM-MSCs were resuspended in the above-mentioned volumes of thrombin solution, cell culture medium, and sealer protein solution and cultured for 2 days at 37°C under 5% CO_2_. Concurrently, BM-MSCs under standard culture conditions served as a positive control of cell viability, whereas the mixture of thrombin solution and “sealer protein solution,” in combination, was used as the negative control.

### Animals, Experimental Design, Anesthesia, Analgesia, and Ultrasonography

**Figure 1:**
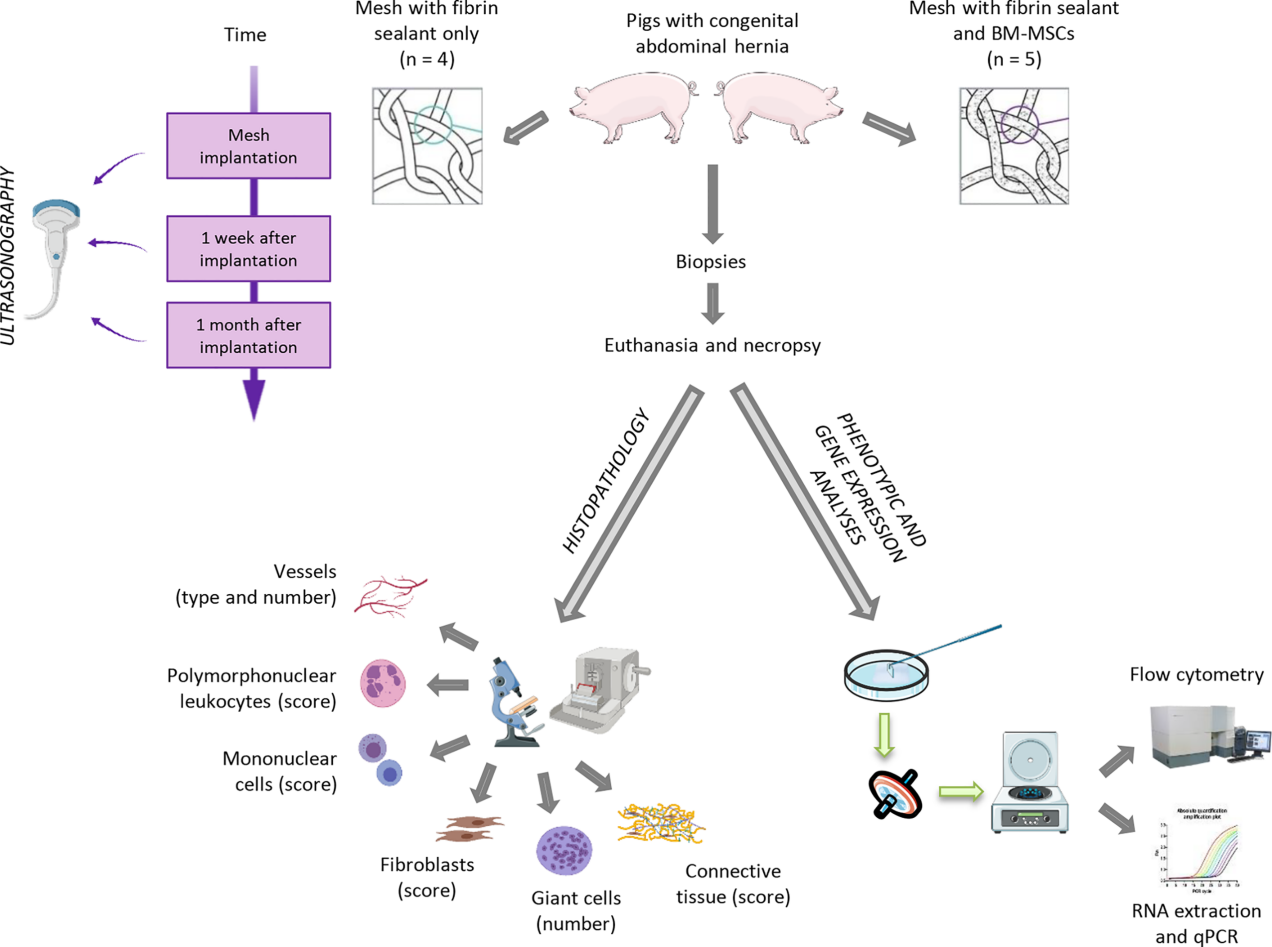
Overview of the experimental approach. Large White pigs with a congenital abdominal non-incarcerated hernia were randomly divided into “control” and “cell” groups. Polypropylene surgical meshes were covered with a fibrin sealant solution (control group) or with porcine bone marrow-derived mesenchymal stem cells (BM-MSCs) (cell group) and were implanted into the animals by laparoscopy. All animals were then biopsied (1 week after implantation) and euthanized prior to the excision of the surgical mesh (1 month after implantation). Prior to all surgeries, an ultrasonographic examination of the hernia was carried out to record the maximal diameter of the hernia orifice. Flow cytometry and histological and gene expression analyses of the biopsy and necropsy samples were performed. qPCR, quantitative real-time polymerase chain reaction. Mesh images are taken from Marinaro et al., 2019. The entire figure has been created with BioRender (https://app.biorender.com/) and Smart Servier Medical Art (https://smart.servier.com/).

The experimental approach that we used is shown in **Figure 1**. The number of animals for our pilot study was defined and approved by the Ethics Committee on Animal Experiments of JUMISC. All the experimental surgical procedures were performed on 10 Large White pigs that initially weighed 38.9 ± 11.2 kg and had a congenital abdominal non-incarcerated hernia. These animals were randomly divided into control (*n* = 5) and cell (*n* = 5) groups and underwent three surgeries at different times: mesh implantation (day 0), biopsy (day 6 or 7, hereinafter referred to as 1 week after implantation), and euthanasia and necropsy (days 28–31, hereinafter referred to as 1 month after implantation). Prior to the surgical procedures, all animals were administered with 0.3 mg/kg diazepam and 20 mg/kg ketamine intramuscularly. Anesthesia induction was achieved with 2–3 mg/kg propofol administered intravenously and maintained with 2.3–2.5% sevoflurane. After each surgery, all animals were administered with 0.01 mg/kg buprenorphine, 0.2 mg/kg meloxicam, and 15 mg/kg amoxicillin. One month after the implantation, anesthesia was induced in all animals as previously mentioned; they were then euthanized with intravenous administration of 1 mEq/kg of KCl.

**Figure 2:**
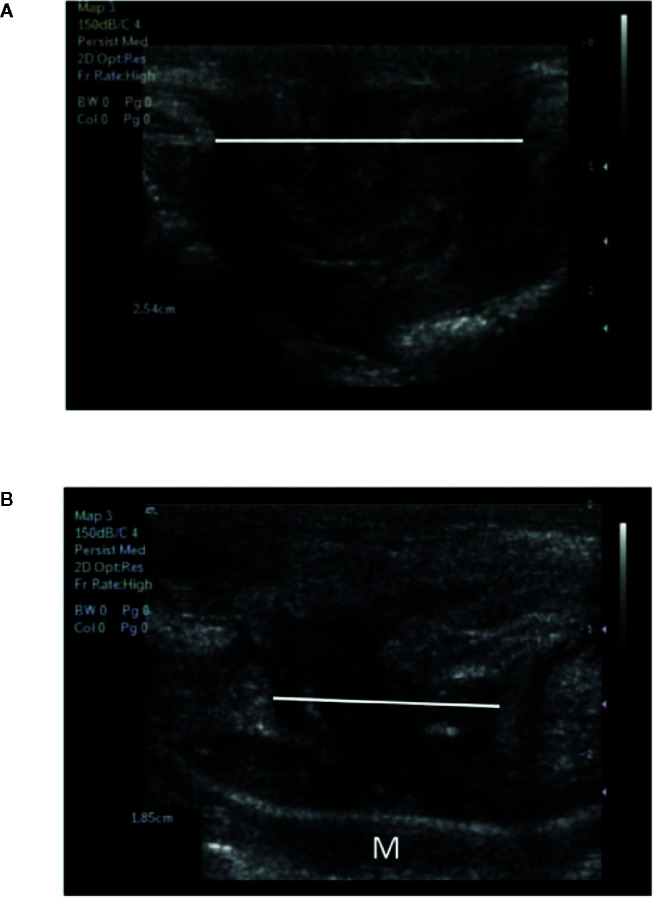
Appearance of the congenital hernia using ultrasonographic imaging before and after mesh implantation surgery. **(A)** Ultrasonographic evaluation before surgical mesh implantation (maximal diameter of the hernia 2.54 cm). **(B)** Ultrasonographic findings 1 month after mesh implantation surgery. A decrease in the diameter of the hernia can be observed (maximal diameter of the hernia 1.85 cm). Implanted mesh (M) appears as a linear echogenic interface.

Prior to all the implantation surgeries, an ultrasonographic examination of the hernia was performed on each animal by an experienced operator (FD), and the maximal diameter of the hernia orifice was recorded according to the short-axis view (**Figure 2**). To describe the evolution of the ultrasonographic findings and the hernia measurements after mesh implantation surgery, an ultrasonographic assessment was performed 1 week and 1 month after implantation. The results are presented in terms of percent reduction.

### Mesh Preparation and Laparoscopic Surgery Procedures for Hernia Repair

Monofilament polypropylene (PP) meshes (90 g/m^2^ weight; Assumesh^®^, Assut Europe, Italy) were cut into 6 × 6 cm pieces and used for the surgical repair of abdominal hernias in both groups. For the cell group, allogeneic BM-MSCs were detached from flasks with 0.25% trypsin solution and stained with Trypan Blue stain (0.4%) (Thermo Fisher Scientific, Waltham, MA, USA) and counted with a Countess^®^ Automated Cell Counter (Thermo Fisher Scientific, Waltham, MA, USA). A Trypan Blue dye exclusion test showed a viability of more than 95%. A total of 9 × 10^6^ cells were resuspended in 3 ml of a 3:1 ratio of complete cell culture medium to thrombin solution (according to the results of the previous clotting and cell viability assays). This cell suspension was then mixed with 3 ml sealer protein solution and applied on the top of each PP mesh by using the fibrin sealant Tisseel^®^ applicator.^2^ As previously stated, the thrombin solution (500 IU/ml thrombin) and sealer protein solution (91 mg/ml fibrinogen) were provided with the commercially available fibrin sealant Tisseel^®^ (Baxter). Cell dose was based on one of our previous studies (Blázquez et al., 2018) and optimized according to mesh size, the minimum volume of fibrin sealant to obtain complete coverage of the mesh surface, and the potential cell loss due to laparoscopic handling.

For the control group, the same volumes of complete cell culture medium, thrombin solution, and sealer protein solution without cells were mixed and spread on top of the PP meshes by using the fibrin sealant Tisseel^®^ applicator. The approximation of hernia borders was performed through intracorporeal suturing by expert laparoscopic surgeons (FS-M and MB). The previously prepared meshes for the control and cell groups were carefully rolled inside a trocar for laparoscopic implantation. The surgical implantation was performed through laparoscopy by using 8–10 helicoidal staples.

**Figure 3:**
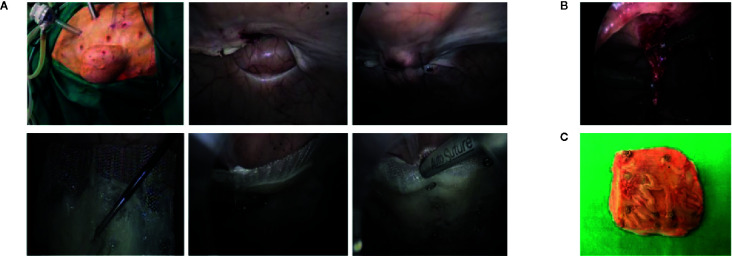
Surgical approach. **(A)** The abdomen of the pig was insufflated with CO_2_ and the hernia orifice was visualized from the outside and from the inside with a laparoscopic camera. Hernia borders were approximated by intracorporeal suturing. The rolled surgical meshes (prepared as shown in **Supplementary Figure 1**) were inserted into a trocar and placed in contact with the hernia. Then the mesh was fixed with helicoidal staples. **(B)** Small biopsy samples of the mesh and its surrounding tissues were collected with Metzenbaum scissors 1 week after implantation surgery. **(C)** The meshes were excised following euthanasia of the animals 1 month after the implantation.

A week after implantation, laparoscopic inspections were performed and small biopsy samples of the mesh with its surrounding muscle–peritoneum were collected with Metzenbaum scissors for further analyses. A month after implantation, the animals were euthanized and macroscopically evaluated. The surgically implanted meshes were excised from the euthanized animals and samples were taken for histology, flow cytometry, and gene expression analyses. Representative images of the surgical procedures are shown in **Figure 3**.

### Histological Analysis

**Figure 4:**
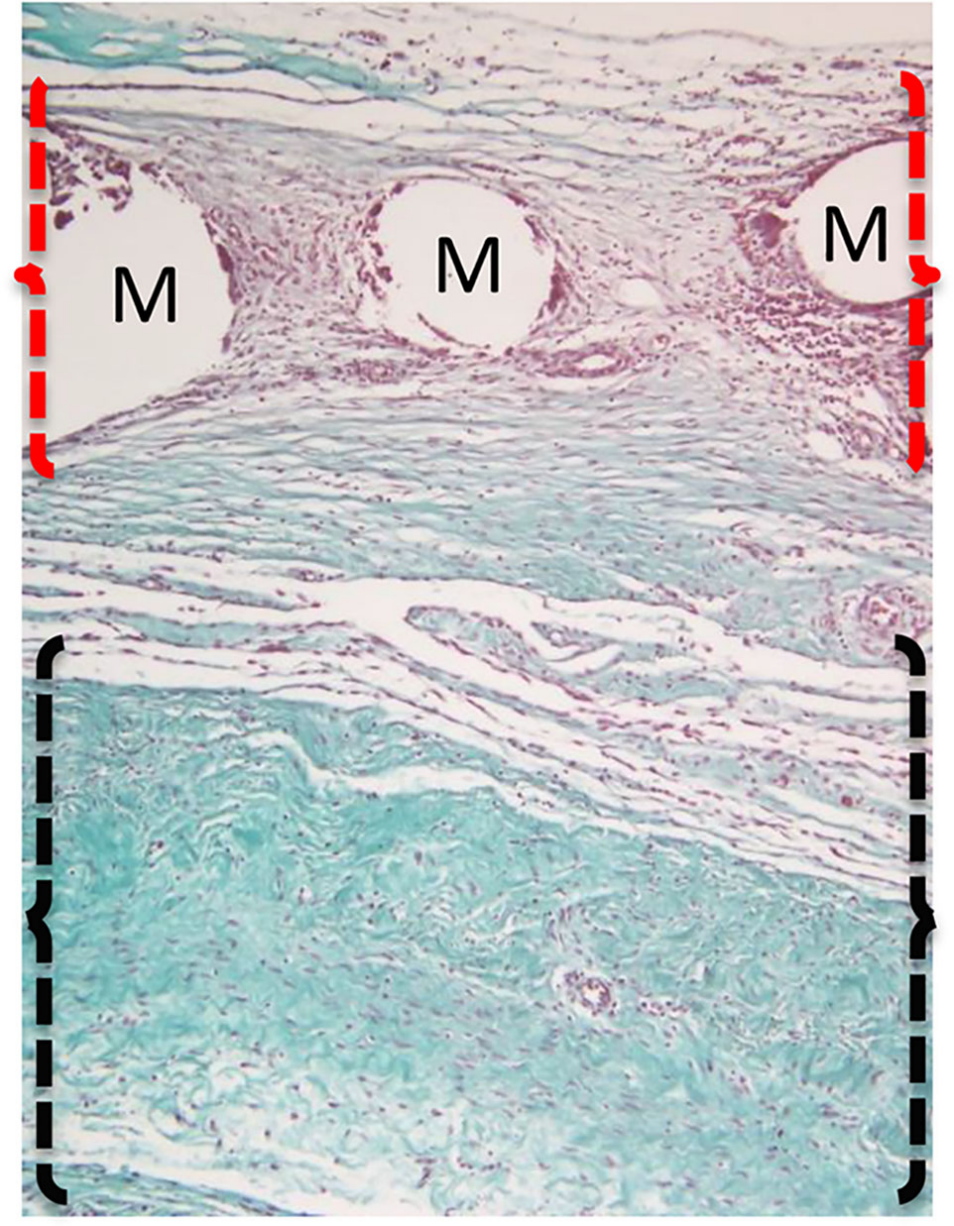
Representative image of a surgically implanted mesh and its surrounding tissue. The assessment of cellular characteristics was performed in the mesh (M) area (indicated by red brackets), whereas the connective tissue was evaluated in the area below the mesh (indicated by black brackets). The image represents a tissue sample stained with Masson’s trichome stain.

The samples obtained 1 week and 1 month after implantation were washed with PBS to remove excess blood, and histological analysis of the whole layer composed of the mesh and muscle–peritoneum was performed. All the histological samples were fixed in 4% formaldehyde, embedded in paraffin, sliced into 5–8 µm thick sections for histological analysis, and stained with hematoxylin and eosin (HE) and Masson’s trichrome (MT). The microscopic evaluation of the specimens was performed on the tissue area where the mesh was implanted (clear circular areas representing mesh fibers) except for the connective tissue, which was also assessed below the mesh (**Figure 4**). The histological features, except the number of giant cells, were evaluated and counted in five fields distributed along the length of the specimen (oil immersion objective). Each specimen was first evaluated under low magnification in order to exclude necrotic or less preserved areas. Mononuclear and polymorphonuclear cells and fibroblasts were counted and assigned scores (**Table 2**) according to their mean number in the five fields of the HE specimens. Vessels (HE specimens) and connective tissue (MT specimens) were grouped according to their appearance and assigned scores (**Table 2**). The scores (**Table 2**) were assigned according to a previously published score system (Badylak et al., 2002). Vessels and giant cells (HE specimens) were counted and recorded according to their mean number in the five oil immersion magnification fields. Moreover, the average number of giant cells around mesh fibers was also determined under high-power field (×40 objective) and recorded.

**Table 2:** Scoring system for histological evaluation of the tissue surrounding and infiltrating the surgical meshes 1 week and 1 month after implantation.

Parameter	Score				
	0	1	2	3	4
Polymorphonuclear leukocytes	No cells	Between 0 and 5 cells	Between 6 and 10 cells	Greater than 10 cells	
Mononuclear cells	No cells	Between 0 and 5 cells	Between 6 and 10 cells	Greater than 10 cells	
Vessels	No vessels	1–3 blood vessels	4–10 blood vessels	Greater than 10 blood vessels	
Vessel type	No vessels	Small arterioles and/or small venules and/or capillaries	Arterioles and/or venules	Both types	
Fibroblasts	No cells	Between 0 and 5 cells	Between 6 and 10 cells	Greater than 10 cells	
Connective tissue	No connective tissue	Loose (areolar) connective tissue with sparse and random arrangement of fibers	Moderately dense connective tissue with increased number of collagen fibers	Dense irregular (non-organized) connective tissue enriched with collagen fibers but the fibers are randomly arranged	Dense regular or organized connective tissue enriched with collagen fibers and the fibers tend to be organized in parallel bundles

The scores were adapted from a previously published score system (Badylak et al., 2002). The score for each parameter was evaluated per oil immersion field (×100 objective).

### Flow Cytometry and Quantitative Polymerase Chain Reaction Studies

Phenotypic and gene expression analyses were performed on the infiltrating cells in the implanted surgical meshes. To collect these cells, the samples obtained 1 week and 1 month after implantation were washed with PBS to remove blood and other residues, and the muscle–peritoneum was removed. The meshes were moved to Petri dishes, submerged in PBS, and scraped with a blade to collect the outer layer of cells. The PBS containing the scraped cells was collected and filtered through a 40 µm filter (Fisher Scientific, Leicestershire, UK) to remove debris and the PP filaments. Afterwards, the infiltrating cells were detached twice from the scraped meshes with 0.25% trypsin solution.

For phenotypic analysis, 2 × 10^5^ cells were resuspended in PBS containing 2% FBS and stained with the appropriate concentrations (according to the manufacturer’s instructions) of fluorescence-labeled monoclonal antibodies against extracellular porcine surface markers T-cell surface antigen T4/Leu-3 (CD4), T-cell surface glycoprotein CD8 alpha chain (CD8α), CD45, Neural cell adhesion molecule 1 (CD56), Scavenger receptor cysteine-rich type 1 protein M130 (CD163) (BD Pharmingen, CA, USA), Monocyte differentiation antigen CD14 (CD14), Low affinity immunoglobulin gamma Fc region receptor III (CD16), CD27, CD45 antigen isoform RA (CD45RA), and SLA-II (Bio-Rad, CA, USA) for 30 min at 4°C (**Table 3**). The cells were washed, resuspended in PBS, and analyzed by using a FACSCalibur™ Flow Cytometry System (BD Biosciences, San Jose, CA, USA). After the acquisition of 10^5^ events, cells were selected according to forward and side scatter characteristics, and fluorescence was analyzed by using CellQuest software (BD Biosciences). Appropriate isotype-matched negative control antibodies were used in all experiments.

**Table 3:** Combination of antibodies used for phenotypic analysis by flow cytometry of the infiltrated cells inside the surgical mesh.

Flow cytometry: combination of antibodies	
Percentage of lymphocyte subpopulations	CD4, CD8, CD16, CD56
Lymphocyte differentiation	CD4, CD8, CD27, CD45RA
Lymphocyte subsets: activation markers	CD4, CD8, CD16, CD56, SLAII
Macrophage infiltration and activation	SLAII, CD14, CD163

The gene expression of cells that infiltrated the surgical meshes 1 week and 1 month after implantation was analyzed by using quantitative real-time polymerase chain reaction (qPCR). The total RNA from scraped and detached cell samples was isolated by using a mirVana™ miRNA Isolation Kit (Invitrogen, Thermo Fisher Scientific Inc., Waltham, MA, USA) according to the manufacturer’s instructions. RNA quality and concentration were spectrophotometrically evaluated by using a Synergy™ Mx Microplate Reader (Biotek, Winooski, VT, USA). Only the RNA samples with a 260/280 nm absorbance ratio between 1.8 and 2.1 were retrotranscribed to complementary DNA (cDNA) and amplified by qPCR. The amount of total RNA required for the reverse transcription reaction was calculated according to their concentrations after isolation such that the same starting cDNAs for qPCR amplifications of the compared study groups was guaranteed (control and cell groups 1 week after implantation; and control and cell groups 1 month after implantation). To this aim, 300 ng cDNA for the 1 week after implantation samples and 700 ng cDNA for the 1 month after implantation samples were synthesized from total RNAs in reverse transcription reactions by using iScript Reverse Transcription Supermix (BioRad, Hercules, CA, USA) with a reaction set-up and thermal cycling protocol according to the manufacturer’s instructions. qPCR was performed by using TaqMan^®^ Gene Expression Assays (Applied Biosystems, Thermo Fisher Scientific Inc., **Supplementary Table 1**) in combination with TaqMan Fast Advanced Master Mix (Applied Biosystems, Thermo Fisher Scientific Inc.). The thermal cycling conditions were as follows: 50°C for 2 min, 95°C for 2 min, and then 40 cycles of 95°C for 1 s and 60°C for 20 s. The amplification of cDNAs was performed by using a QuantStudio 3 System (Applied Biosystems, Thermo Fisher Scientific) and the qPCR products were quantified by a fluorescent method using the 2^-ΔCt^ expression (Livak and Schmittgen, 2001). The duplicates of all samples were analyzed separately and normalized against the *HRPT1* gene. Duplicate no-template control samples were prepared for each gene and showed no DNA contamination.

### Statistical Analysis

The data were statistically analyzed using SPSS-21 software (SPSS, Chicago, IL, USA). For ultrasonographic data, ANOVA and the Tukey test were applied. The normal distribution of variables was assessed with the Shapiro–Wilk test, and the Levene test was used to assess homoscedasticity. For variables with normal distribution and homogeneity of variances, we used Student’s *t*-test, and the Mann–Whitney *U*-test was used for non-parametric and heteroscedastic variables. A *p* < 0.05 was considered statistically significant.

## Results

### Phenotypic Analysis and Multipotentiality of Mesenchymal Stem Cells, Admixture With Fibrin Sealant, Fibrin Clotting, and Cell Viability Assay

The stemness markers expression profile of BM-MSCs was CD29^+^/CD44^+^/CD45^-^/CD90^+^/CD105^+^/SLA-1^+^/SLA-2^–^. Moreover, the differentiation assays assessing adipogenic, chondrogenic, and osteogenic lineages demonstrated the multipotentiality of BM-MSCs; this is consistent with a previously published study (Casado et al., 2012).

Assessment of the clotting capability of the solutions prepared by mixing complete cell culture medium, thrombin solution, and sealer protein solution revealed that clotting took place with any mixture containing thrombin solution with volumes up to 25 µl. Less liquid leakage was observed with higher volumes of thrombin solution (data not shown).

The cell viability CCK-8 assay demonstrated the highest cell viability when the complete cell culture medium-to-thrombin solution ratio was 3:1 (data not shown). Hence, the latter ratio of complete cell culture medium-to-thrombin solution was: (i) mixed with the same volume of sealer protein solution and used to coat the PP mesh before implantation surgery (control group) or (ii) used to prepare the MSC suspension, mixed with the same volume of sealer protein solution, and used to coat the PP mesh (cell group).

### Evaluation of Congenital Hernia Size

**Figure 5:**
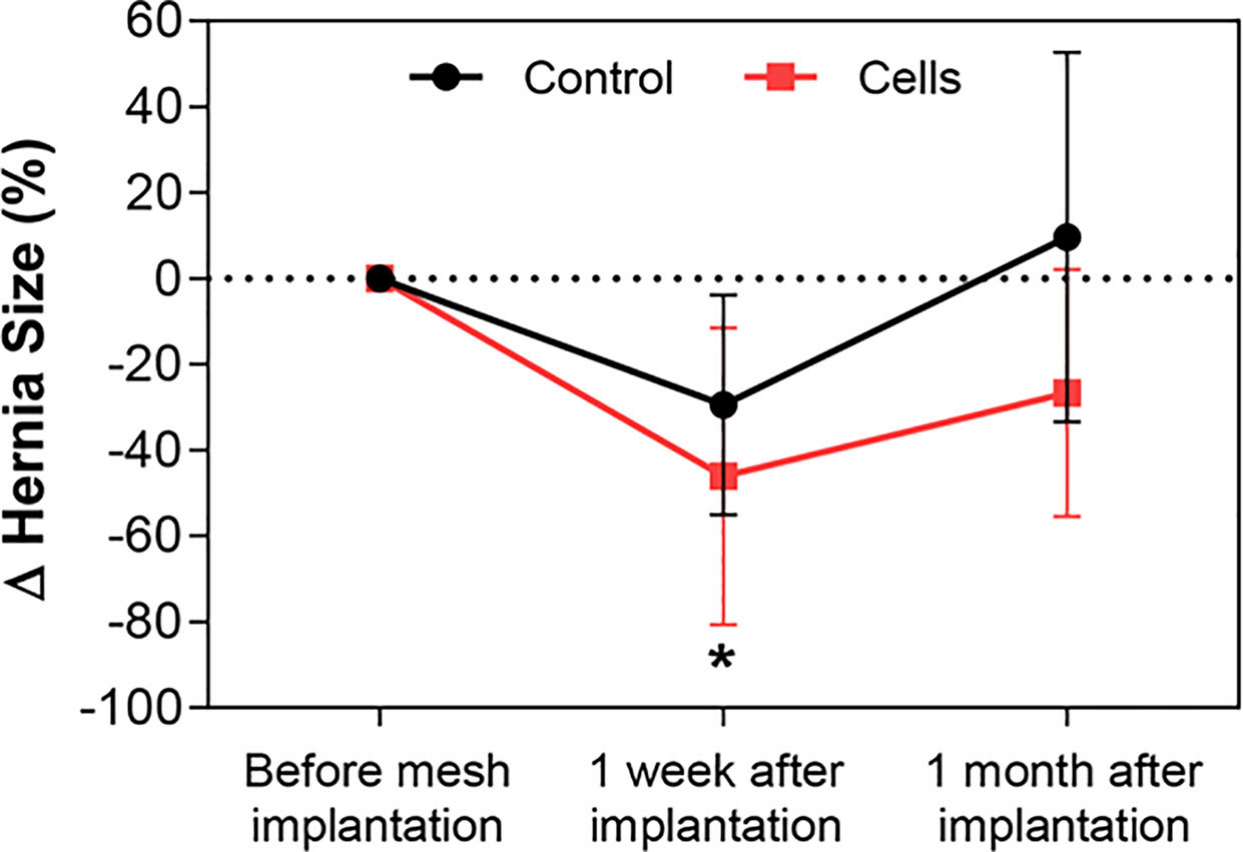
Evaluation of hernia size by ultrasonography. The maximum diameter of the hernia orifice was recorded by ultrasonography before, 1 week after, and 1 month after hernia border approximation and mesh implantation surgery in all animals in the two groups: control (black line) and cell group (red line). Changes in hernia size are presented as percent reduction at different time points, with the initial size of the hernia pertaining to the size before mesh implantation. A statistically significant decrease in the size of the hernias was observed 1 week after implantation in the cell group compared with the size recorded before mesh implantation surgery. All data are presented as mean ± standard deviation. The graph was created with GraphPad Prism. *p < 0.05 refers to the size of the hernia before mesh implantation.

Ultrasonographic assessment of hernia size is presented in terms of percent reduction of the mean hernia size 1 week and 1 month after implantation and compared with the hernia size before the suturing of hernia borders and mesh implantation (implantation day). The mean size of the congenital hernias before mesh implantation was 2.49 ± 0.99 cm (0%). As shown in **Figure 5**, the approximation of the hernia borders by suturing reduced the hernia size by 29.49 ± 25.72% 1 week after implantation and increased the same by 9.58 ± 43.15% 1 month after implantation in the control group, with reference to the size on implantation day. The cell group had a reduction of 46.01 ± 34.69% 1 week after implantation and a further reduction of 26.61 ± 28.79% 1 month after implantation, with reference to the size on implantation day. The decrease in the mean size of the hernia 1 week after implantation in the cell-treated group compared with the mean size of the hernia on implantation day was statistically significant (*p *< 0.05).

### Surgical Mesh Implantation by Laparoscopic Surgery

The laparoscopic procedures allowed successful mesh implantation in all animals. In most cases (7 out of 10 animals), the implantation site did not show excessive inflammation or tissue adhesions except for three pigs that had adherence of the omentum, spleen, and small intestine to the surgical mesh. One animal showed hernia maintenance and another had *Escherichia coli* infection of the peritoneum and implant site. One pig manifested anorexia and vomiting 2 days after mesh implantation and died 4 days after implantation. Unfortunately, it was not possible to determine whether the cause of death was associated with mesh implantation surgery. The mean duration of the surgical procedure for hernia implantation was 41.27 ± 15.18 min per animal. Our results demonstrated that the surgical procedure, fixation method, and treatments were well tolerated and feasible to perform in this animal model. Additionally, considering that safety is one of the major issues in stem cell-based therapies, this animal model was useful in determining the hypothetical adverse effects of MSCs admixed with a fibrin sealant. Macroscopic evaluation of the incisional hernia and implanted meshes 1 week and 1 month after implantation showed a normal morphology of the tissues. Surgical adhesions, effusions, or tissue fibrosis were not observed in any of the groups.

### Histological Evaluation of the Mesh Implant Site

**Figure 6:**
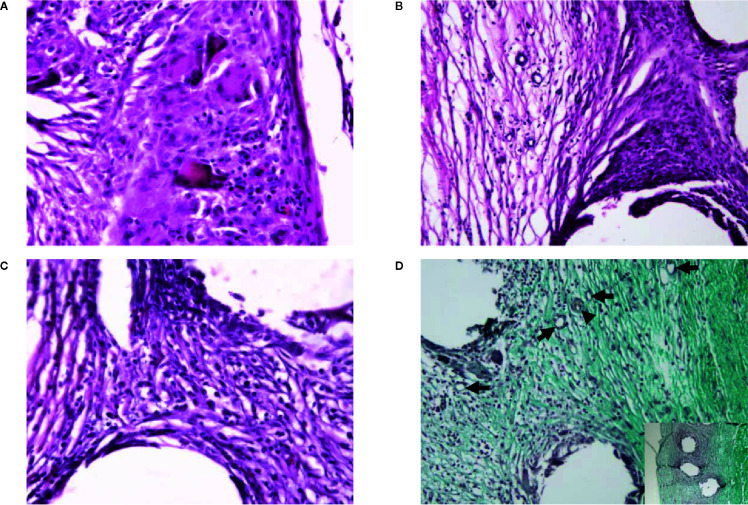
Representative figures of the histology of a control case and a cell case 1 month after implantation. **(A)** Polymorphonuclear cells around the mesh, along with inflammatory giant cells in a control case. Hematoxylin–eosin, ×40 objective. **(B)** Highly cellular connective tissue between the mesh with few collagen fibers below the mesh area in a control case. Hematoxylin–eosin, ×20 objective. **(C)** Presence of mononuclear infiltrate with few polymorphonuclear cells around the mesh in a cell case. Hematoxylin–eosin, ×40 objective. **(D)** Moderately dense connective tissue-enriched blood vessels (*arrows*; including arteriole, *arrowhead*) with some organization of the collagen fibers is present between the mesh fibers in a cell case. Note the dense organized connective tissue with parallel bundles of fibers below the mesh (*inset*). Masson Trichrome, ×20 objective and ×10 objective (*inset*).

**Figure 7:**
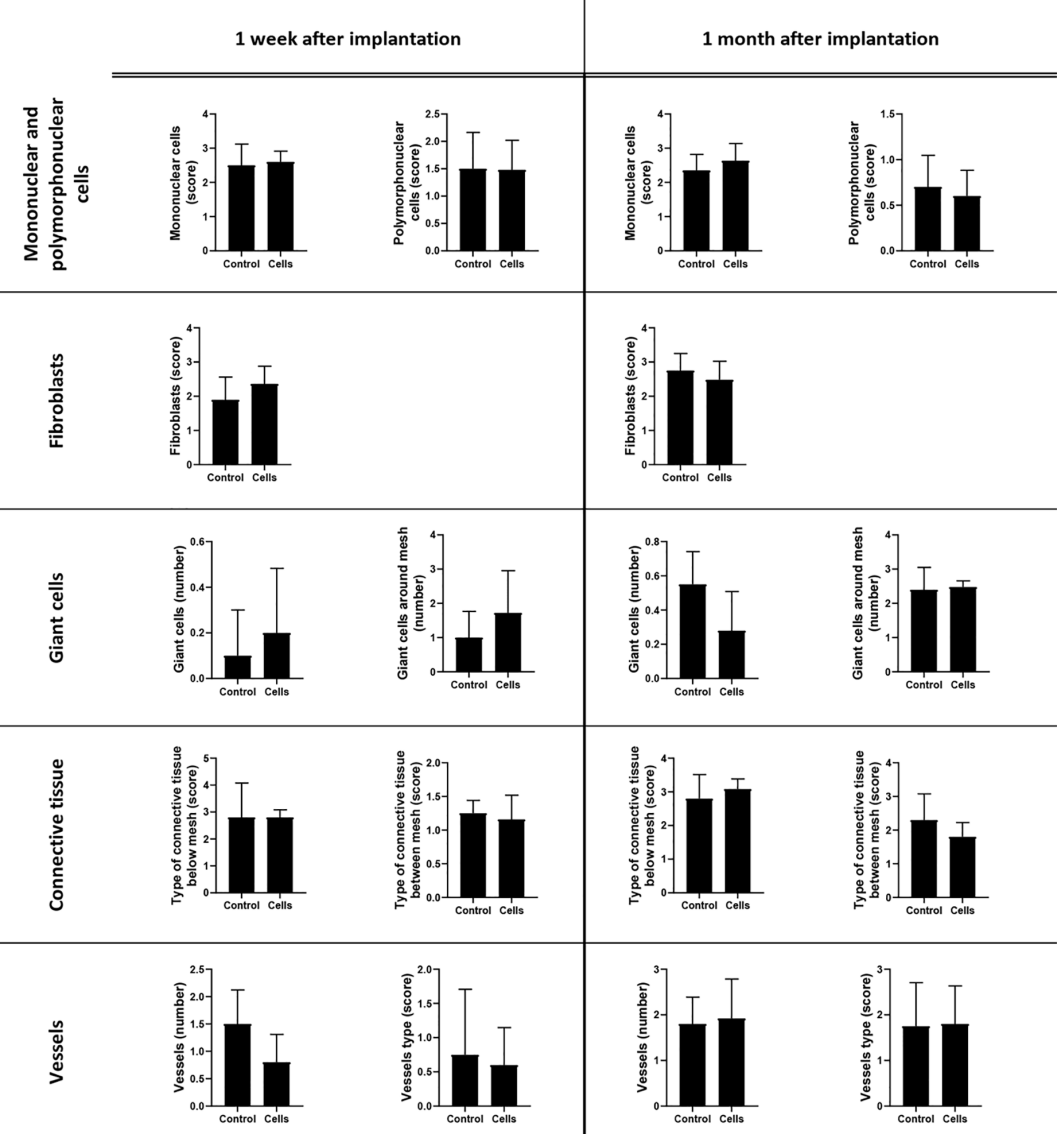
Histological evaluation of surgical meshes and surrounding tissues 1 week and 1 month after mesh implantation. The microscopic appearance of the tissue surrounding the surgical mesh was examined in the hematoxylin–eosin (HE) and Masson’s trichome-stained specimens. Polymorphonuclear leukocytes (score), mononuclear cells (score), vessels (type and number), giant cells (number), and fibroblasts (score) between the mesh area were evaluated in five oil immersion fields. The type and organization of the connective tissue presented between and below the mesh were also scored. The average number of giant cells (number around the mesh fibers) was evaluated under a high-power field (×40 objective field). The scoring criteria are shown in **Table 2**. All the data are presented as mean ± standard deviation. The graphs were created with GraphPad Prism 8.

Histological evaluation of the cellular characteristics in the mesh area was performed. Connective tissue was observed also below the mesh area (**Figure 4**). Histological samples from the control group 1 month after implantation showed the presence of polymorphonuclear and inflammatory giant cells around the mesh area, whereas samples from the cell group 1 month after implantation showed a mononuclear infiltrate with few polymorphonuclear cells around the mesh. Regarding the connective tissue, the control group showed highly cellular connective tissue between the mesh threads, with few collagen fibers below the mesh area 1 month after implantation. In contrast, a moderately dense connective tissue enriched in blood vessels with some organization of the collagen fibers could be observed in the mesh area in the cell group. Additionally, a dense organized connective tissue with parallel bundles of fibers was seen below the mesh in the cell group 1 month after implantation (**Figure 6**). Nevertheless, when the histological features were counted and their scores were compared, no statistically significant differences were observed among the groups. The tissue and cellular characteristics underneath and between the mesh fibers did not seem to be affected by the presence of BM-MSCs at either 1 week or 1 month after implantation (**Figure 7**).

### Phenotypic Evaluation of Cells Infiltrating the Surgical Mesh

Apart from the histological findings, our study also identified and characterized mesh-infiltrated cells. This analysis was performed by using flow cytometry of tissue obtained by biopsy 1 week after implantation and in explanted surgical meshes 1 month after implantation. Flow cytometry of the mesh-infiltrated cells showed an increase in tissue-infiltrated CD14^+^CD163^+^ (M2 macrophages) when the cell group was compared with the control group 1 week after implantation, but this change was not significant. The geometric mean of the activated macrophages in the cell group (52.96 ± 5.42%) significantly decreased 1 month after implantation (*p *< 0.05) compared with that of the control group (80.39 ± 19.55%). The results of the phenotypic analysis by flow cytometry are reported in **Tables 4**, **5**.

**Table 4:** Results of the phenotypic analysis by flow cytometry of the infiltrated lymphocytes and macrophages inside the surgical meshes 1 week and 1 month after implantation surgery.

					1 week after implantation		1 month after implantation	
					Control	Cells	Control	Cells
**Tissue-infiltrating lymphocyte subsets**	**T-helper cells**	**Differentiation phenotype**	Effector-memory cells (%CD45RA^–^/CD27^–^on CD4^+^ CD8α^–^)		87.4 ± 3	88.42 ± 8.83	84.23 ± 3.37	83.49 ± 5.11
			Naive cells (%CD45RA^+^ or CD27^+^ on CD4^+^ CD8α^–^)		12.59 ± 3	11.58 ± 8.83	15.76 ± 3.37	16.5 ± 5.11
		**Activation markers**	NK-related receptor (%CD56/CD16^+^ on CD4^+^ CD8α^–^)		23.48 ± 13.37	12.18 ± 8.63	16.17 ± 10.3	9.08 ± 2.39
			SLA-2 receptor (%SLA-2^+^ on CD4^+^ CD8α^–^)		37.28 ± 22.77	24 ± 14.25	23.07 ± 11.39	18.2 ± 4.77
	**T-cytotoxic cells**	**Differentiation phenotype**	Effector-memory cells (%CD45RA^–^/CD27^–^ on CD4^–^ CD8α^+^)		44.55 ± 19.99	68.65 ± 13.18	51.61 ± 12.53	60.78 ± 9.09
			Naive cells (%CD45RA^+^ or CD27^+^ on CD4^–^ CD8α^+^)		55.44 ± 19.99	31.34 ± 13.18	48.39 ± 12.53	39.21 ± 9.09
		**Activation markers**	NK-related receptor (%CD56/CD16^+^ on CD4^–^ CD8α^+^)		17.98 ± 11.83	15.05 ± 5.89	16.43 ± 11.48	13.67 ± 12.13
				SLA-2 receptor (%SLA-2^+^ on CD4^–^ CD8α^+^)	56.59 ± 25.61	51 ± 18.33	19.79 ± 11.81	22.37 ± 19.01
**Tissue-infiltrating macrophage subsets**	**M1/M2 phenotype**			M2 macrophages (%CD163^+^ on CD14^+^)	67.3 ± 11.72	72.56 ± 4.22	82.42 ± 14.93	83.82 ± 5.99
				M1 macrophages (%CD163^–^ on CD14^+^)	32.69 ± 11.72	27.44 ± 4.22	17.57 ± 14.93	16.17 ± 5.99
	**Activation markers**			SLA-2 expression (SLA-2 geometric mean on CD14^+^)	73.49 ± 20.07	77.41 ± 22.79	80.39 ± 19.55	52.96 ± 5.42*

All data are presented as the mean ± standard deviation. *p < 0.05 refers to the control group. CD4, T-cell surface antigen T4/Leu-3; CD8α, T-cell surface glycoprotein CD8 alpha chain; CD14, Monocyte differentiation antigen CD14; CD16, Low affinity immunoglobulin gamma Fc region receptor III; CD45RA, CD45 antigen isoform RA; CD56, Neural cell adhesion molecule 1; CD163, Scavenger receptor cysteine-rich type 1 protein M130; SLA-2, Swine leukocyte antigen class 2.

**Table 5:** Results of the phenotypic analysis by flow cytometry of the infiltrated leukocytes inside the surgical meshes 1 week and 1 month after mesh implantation.

		1 week after implantation		1 month after implantation	
		Control	Cells	Control	Cells
**Tissue-infiltrating leukocytes**	T-helper cells (% CD4^+^ CD8α^–^)	4.39 ± 2.73	15.67 ± 11.01	12.71 ± 12.31	2.25 ± 1.65
	T-cytotoxic cells (% CD4^–^ CD8α^+^)	10.71 ± 6.51	28.48 ± 15.08	17.46 ± 18.42	2.38 ± 1.17
	Ratio CD4^+^:CD8α	1.16 ± 0.96	0.55 ± 0.2	0.95 ± 0.55	0.88 ± 0.26
	NK cells (% CD8α^–^ CD16^+^/CD56^+^)	5.04 ± 4.68	17.86 ± 14.96	8.69 ± 7.49	4.11 ± 0.49
	Macrophages (% CD14^+^)	14.41 ± 22.45	16.32 ± 12.19	10.23 ± 9.01	5.56 ± 1

All data are presented as mean ± standard deviation.

### Gene Expression Analysis of Cells Infiltrating the Surgical Mesh

**Figure 8:**
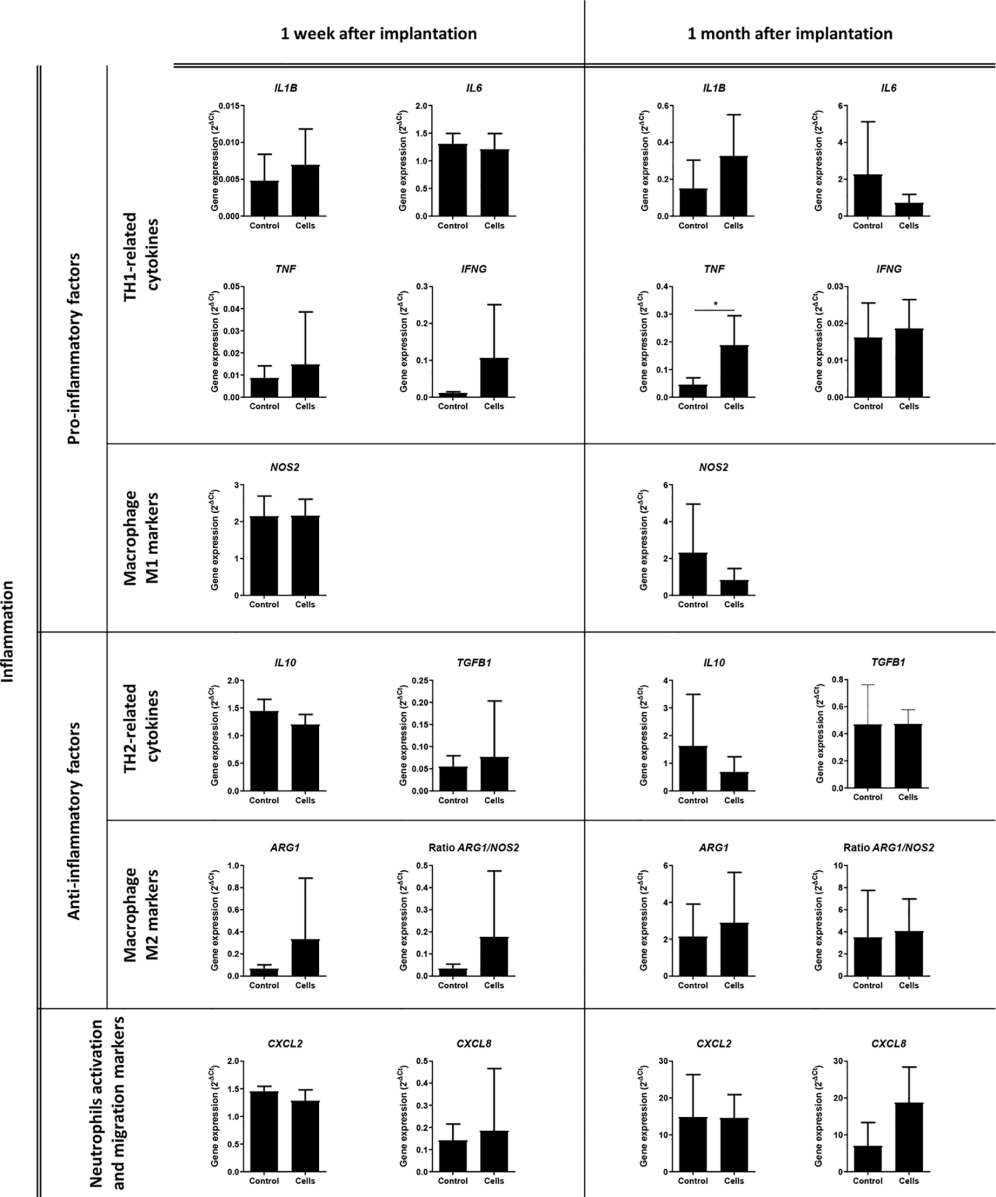
Analysis of the expression of inflammation-related genes. Quantitative real-time polymerase chain reaction was performed on the infiltrated cells within the surgical meshes 1 week and 1 month after mesh implantation. All the data are presented as mean ± standard deviation. The graphs were created with GraphPad Prism. *p < 0.05 refers to the control group. ARG1, Arginase 1; CXCL2, C-X-C Motif Chemokine Ligand 2; CXCL8, C-X-C Motif Chemokine Ligand 8; IFNG, Interferon Gamma; IL1B, Interleukin 1 Beta; IL6, Interleukin 6; IL10, Interleukin 10; NOS2, Nitric Oxide Synthase 2; TGFB1, Transforming Growth Factor Beta 1; TNF, Tumor Necrosis Factor.

**Figure 9:**
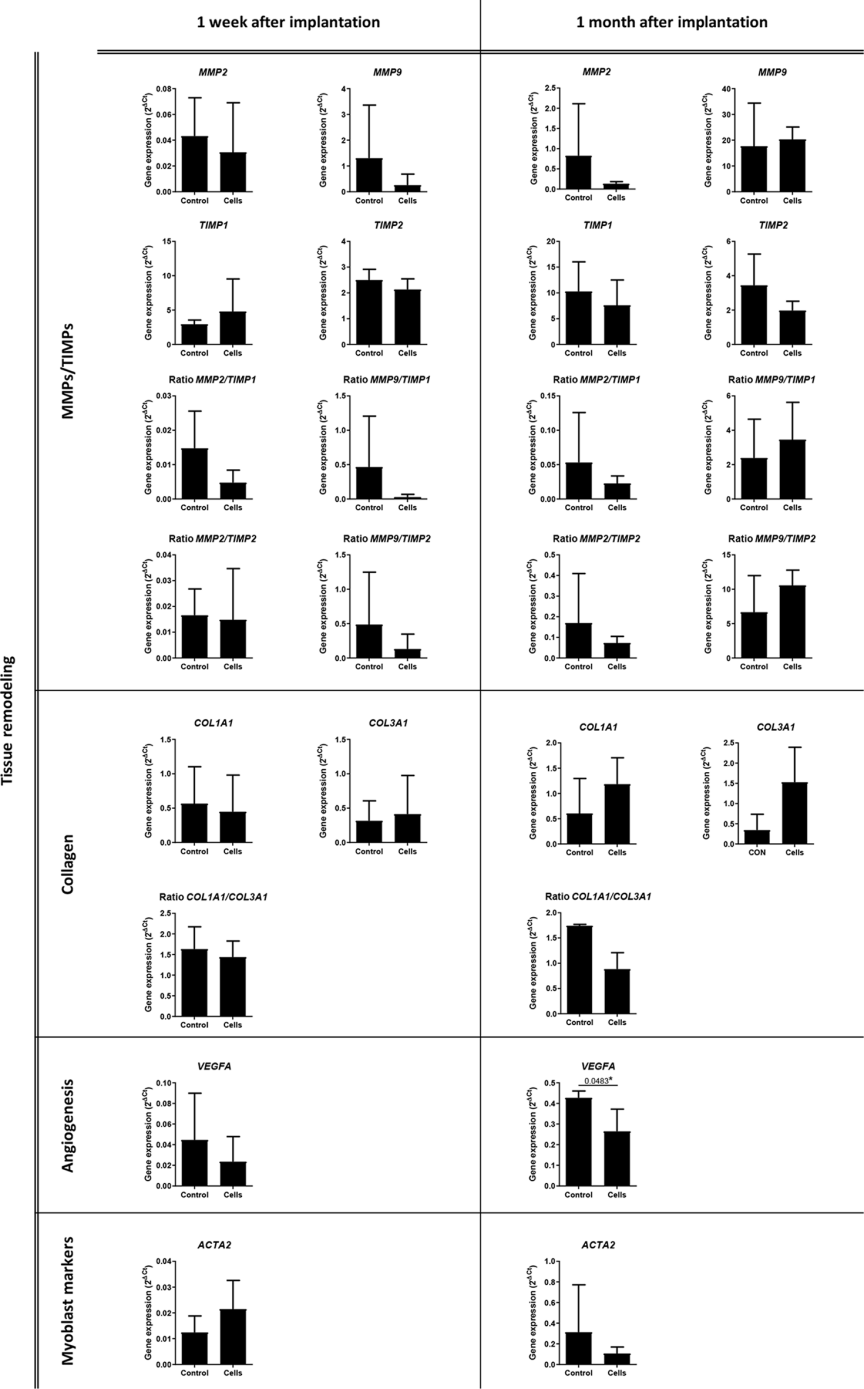
Analysis of the expression of tissue remodeling-related genes. Quantitative real-time polymerase chain reaction was performed on the infiltrated cells within the surgical meshes 1 week and 1 month after mesh implantation. All the data are presented as mean ± standard deviation. The graphs were created with GraphPad Prism. *p < 0.05 refers to the control group. ACTA2, Actin Alpha 2, Smooth Muscle; COL1A1, Collagen Type I Alpha 1 Chain; COL3A1, Collagen Type III Alpha 1 Chain; MMP2, Matrix Metallopeptidase 2; MMP9, Matrix Metallopeptidase 9; TIMP1, TIMP Metallopeptidase Inhibitor 1; TIMP2, TIMP Metallopeptidase Inhibitor 2; VEGFA, Vascular Endothelial Growth Factor A.

The expression of 32 genes by the cells that infiltrated the surgical meshes was quantified 1 week and 1 month after implantation. **Figures 8**, **9** represent the analysis of gene expression when consistent amplifications were obtained with qPCR at both time points. The decrease in the expression of vascular endothelial growth factor A (*VEGFA*) (control group 0.427 ± 0.033 versus cell group 0.265 ± 0.108, *p *= 0.0483) and the increase in tumor necrosis factor (*TNF*) expression (control group 0.046 ± 0.025 versus cell group 0.188 ± 0.106, *p *= 0.0357) were statistically significant in the cell group 1 month after implantation (*p *< 0.05).

## Discussion

Hernia remains a notable problem in human and veterinary medicine despite the fact that its conventional treatment was proposed for the first time in 1890 (Baylón et al., 2017). As a matter of fact, if surgical meshes had become the standard procedure for repairing abdominal hernias, an adverse inflammatory response would usually be observed after implantation, producing a multitude of complications and side effects.

Adult stem cells can differentiate into a wide variety of cell types (Pittenger, 1999) and can be isolated from different tissues such as liver, lung, adipose tissue, skeletal muscle, amniotic fluid, bone marrow, skin, and heart (Mushahary et al., 2018). They have regenerative properties owing to their ability to differentiate and secrete factors that locally activate progenitor cells (Uccelli et al., 2008). Bearing these properties in mind, different cell-based treatments have been proposed to reduce the adverse inflammation and to improve tissue integration and regeneration after surgical mesh implantation (Marinaro et al., 2019). Nevertheless, it is important to consider that some clinical trials involving stem cell therapy have successfully reached the third phase, where long-term benefits and side effects are evaluated. However, many of them have not yielded the desired results (Trounson and McDonald, 2015) and their number has dropped over the years. This decline may be due to the fact that these preclinical and clinical trials are too heterogeneous: MSCs from different sources, different cell preparation protocols, and different cell passage numbers have been used over time (Kabat et al., 2020). Under these circumstances, the application of stem cell therapy to the surgical implantation of meshes for hernia treatment remains challenging. Even in this particular field, there is a lack of standardization in preclinical trials. Hence, creating a consensus about surgical procedures, the type of surgical meshes to use, and the effectiveness of stem cells in the pathophysiology of hernia is difficult (Marinaro et al., 2019).

First, there is a lack of uniformity regarding the use of animal models in preclinical trials (Vogels et al., 2017). Even the most recent studies that have investigated the use of stem cells on surgical meshes have been performed *in vitro* (Gao et al., 2014; Vozzi et al., 2017) or in small animal models, especially in mice (Darzi et al., 2018; Paul et al., 2019; Mukherjee et al., 2020), rats (Altman et al., 2010a; Altman et al., 2010b; Edwards et al., 2015; Iyyanki et al., 2015; Klinger et al., 2016; van Steenberghe et al., 2017; Hansen et al., 2020), and rabbits (Zhao et al., 2012; Cheng et al., 2017). Only a few preclinical studies have been performed in sheep (Gerullis et al., 2013; Gerullis et al., 2014). However, this animal model has only been used for the study of surgical meshes in the case of pelvic organ prolapse (Emmerson et al., 2019), owing to similarities between the ovine and human urogenital tracts. To date, and to our knowledge, only one clinical case involving a human patient has been published (Palini et al., 2017).

Second, stem cell-based therapies for the treatment of hernia have been developed by using different stem cell sources such as placenta-derived stem cells (Zhang et al., 2016), endometrium-derived MSCs (Su et al., 2014; Ulrich et al., 2014; Edwards et al., 2015; Darzi et al., 2018), and adipose-derived MSCs (Melman et al., 2011; Li et al., 2013; Iyyanki et al., 2015; Blázquez et al., 2016; Cheng et al., 2017).

Third, surgical meshes can be used to reinforce tissues in pelvic prolapses or hernias, but these two conditions are quite different. The most recent and relevant studies investigating the use of stem cells on surgical meshes are focused towards reinforcement of the pelvic floor (Emmerson et al., 2019; Paul et al., 2019; Mukherjee et al., 2020) rather than of the abdominal wall; however, it is necessary to consider the fundamental differences between the two pathological conditions in the evaluation of preclinical trials.

Fourth, even though standardization and reproducibility are very important to obtain consistent results in research, the clinical setting is characterized by a huge variability in patients, with a variety of body masses and type, position, and size of hernias. Preclinical trials should involve animal models resembling the variability of human and veterinary patients to guarantee the safety, feasibility, effectiveness, and applicability of the preclinical results. Most of the studies investigating stem cell-aided surgical mesh hernia repair are performed after a ventral incision (Altman et al., 2010a; Iyyanki et al., 2015; van Steenberghe et al., 2017; Hansen et al., 2020), which may be an appropriate model for incisional hernias following laparotomies but not for other kinds of hernias.

A plethora of different surgical meshes are commercially available; however, they can be generally categorized under three groups: synthetic non-absorbable, synthetic absorbable, and biological meshes (FitzGerald and Kumar, 2014). Each type of mesh may produce different effects on a human or veterinary patient according to its intrinsic characteristics such as material, absorbability, and biocompatibility.

Many surgical meshes for urogynecological use^3^ and for abdominal wall repair^4^ have been withdrawn from the market because of safety concerns. One of the reasons for the recall of surgical meshes may be the lack of thorough understanding in hernia research.

We developed an experimental approach to test whether the use of stem cells for abdominal hernia treatment is viable in a clinically relevant animal model. To the best of our knowledge, this is the first preclinical study where pigs with congenital abdominal hernias were treated with surgical meshes seeded with adult stem cells. Additionally, the surgical approach was performed with minimally invasive procedures to avoid complications related to open surgery. An exhaustive follow-up was performed at different time points using different evaluation methods: ultrasonography, gene expression analysis, complete histological evaluation, and cellular characterization by flow cytometry of infiltrated leukocytes.

Our experimental study was initially focused on the selection of the best animal model. We chose the swine model for different reasons. First, pigs are comparable to humans in terms of body mass, metabolism, organ size, omnivorous diet (Bassols et al., 2014; Schook et al., 2015), and gastrointestinal anatomy (Gonzalez et al., 2015). Second, porcine skin is similar to human skin in different histological and anatomical aspects; for example, the sparse and simple hair coat, epidermal thickness and turnover kinetics, the presence of adipose tissue at the hypodermis, and the presence of musculocutaneous vessels that run perpendicular to the skin’s surface are similar between humans and pigs (Kemppainen, 1990; Avon and Wood, 2005; Debeer et al., 2013; Wei et al., 2017; Fossum and Duprey, 2019). Third, abdominal and inguinal hernias are relatively common in pigs; the incidence of these hernias range from 1.7% to 6.7% in different swine breeds (Atkinson et al., 2017). Piglets frequently present an incomplete closure of the umbilical ring after birth as a result of genetic causes (Grindflek et al., 2018) and are usually rejected by farmers as they have slower growth and higher mortality (Yun et al., 2017).

Hence, in order to evaluate the therapeutic effect of adult stem cells combined with surgical meshes, we chose Large White pigs with congenital abdominal hernias, and two study groups were established: a control group and a cell group. For the application of stem cells in surgical meshes, we considered the synthetic polymer PP as it is chemically inert and does not support cell adhesion. Fibrin sealants allow cell adhesion, viability, migration, and proliferation, and allow cells to execute their paracrine action locally. Moreover, fibrin sealants are rarely related to inflammation and foreign body reaction; hence, they are widely used in tissue engineering (Li et al., 2015). We used a commercially available fibrin sealant to aid cell adhesion on the PP surgical mesh and to aid the compatibility of this cell-seeded mesh with the laparoscopic instrumentation. Finally, we used MSCs that were previously characterized in terms of phenotype, gene expression, and differentiation capacity (Casado et al., 2012; Álvarez et al., 2016) and that were used in preclinical studies without adverse effects (Blázquez et al., 2015). It is important to note that, even though we did not evaluate their clonic capacity, the MSCs used in this study fulfill the “minimal criteria for defining multipotent mesenchymal stromal cells” defined by the International Society for Cellular Therapy (Dominici et al., 2006). Additionally, we proposed the administration of heterologous cells, as they can be safer than autologous cells (Crisostomo et al., 2015). Under these circumstances, all the steps from cell preparation and seeding on the top of the surgical mesh to rolling and insertion within the laparoscopic trocar are easy and quick; the cell-seeded material can be cryopreserved (Blázquez et al., 2018), offering a safe and bioactive off-the-shelf product for hernia repair.

The first aim of this paper was to evaluate the reduction in hernia size after the approximation of hernia borders and the implantation of the surgical mesh by laparoscopy. Additionally, we aimed to test the effect of the stem cells that we seeded on the surgical meshes in the cell group.

Ultrasonography was performed prior to surgical mesh implantation and 1 week and 1 month after mesh implantation surgery. It is important to note that the mean diameter of the congenital hernias in the experimental groups was 2.49 ± 0.99 cm; however, these sizes were heterogeneous and ranged from 0.74 cm to 4.15 cm in diameter. In order to normalize hernia sizes, we presented our results in terms of percent reduction. There was a statistically significant reduction in terms of mean hernia size when surgical meshes were combined with stem cells 1 week after implantation (–46.01 ± 34.69). Obviously, we cannot simply state that this reduction is a cell-mediated effect only as it can be associated with inherent differences in the surgical procedures (different suture closures in different kinds of hernias and subsequent mesh fixation) and with the heterogeneous range of size and weight of the animals in the study.

In both groups, the repair of the hernias and mesh fixation were performed by laparoscopy. Although a systematic review and meta-analysis has revealed that the recurrence rate, infection, hospital stay, and operation time are similar between open surgery and laparoscopy (Al Chalabi et al., 2015), there has not yet been a consensus about the best method to repair ventral hernias (Van Veenendaal et al., 2015). There are reviews and meta-analyses wherein the laparoscopic repair of umbilical hernias was reported to be associated with a lower risk of infections, a lower recurrence rate, and a shorter hospitalization stay (Hajibandeh et al., 2017); based on these and considering that laparoscopy is widely used for hernia surgery, our results have revealed that this surgical procedure is suitable and safe in a swine model. An important advantage of using laparoscopy in the proposed model (animal with hernia congenital disease) is the possibility of evaluating the macroscopic status of internal tissues. Our surgical procedures consisted in the removal of previous adhesions (if present), followed by the closure of the hernial ring with sutures and placement of the mesh. The surgical techniques were successfully executed, even though a reduced number of animals presented some complications. We observed an incomplete closure of the hernial ring in one animal, with no leakage or protrusion of hernial contents. This recurrence of the hernia may have been due to loose sutures or to the intraperitoneal fixation of the mesh with helicoidal staples. We think that the implantation of the helicoidal clips could be insufficient to guarantee deep aponeurotic fixation to support the displacement of tissues during pig growth. We also stated that there was an *E. coli* contamination, which could have been caused by bacterial contamination from a contaminated pneumoperitoneum needle, trocar, or tweezers or by an ineffective antibiotic therapy protocol. Additionally, three animals presented tissue adherences. It is important to note that intraperitoneal implantation places the mesh in direct contact with the visceral peritoneum. This kind of implant *per se* can cause post-surgical adhesions (Farmer et al., 1998). Laparoscopy also allowed us to perform biopsies of the implanted meshes and their surrounding tissues at intermediate time points. In our study, the follow-up was conducted after 1 month, and the biopsies 1 week after implantation allowed us to analyze early histological and genetic changes as well as leukocyte infiltrations at short intervals.

The third aim of this study was to characterize the inflammatory response of the abdominal tissues to surgically implanted PP meshes with or without stem cells. Thus, we evaluated the expression of TH1/TH2 markers and M1/M2 markers in mesh-infiltrated cells by qPCR. We also analyzed mesh-infiltrating leukocytes by flow cytometry and assessed the inflammatory status of the tissue surrounding the surgical meshes through a histopathological examination. Even though previous observations in murine models using MSC-coated meshes (Blázquez et al., 2016; Blázquez et al., 2018) have demonstrated an M2 polarization within the tissue and around the mesh fibers, we did not find any significant change in the expression of M1/M2 markers. Surprisingly, our comparative analyses in 10 different TH1/TH2 cytokines revealed a significant increase in the expression of *TNF* in the cell group 1 month after implantation. An increase in TNF production has already been linked to the implantation of PP meshes in one study (Prudente et al., 2016). Although a reduction in *TNF* gene expression in the cell group was expected (Yan et al., 2018) we hypothesized that the short survival and paracrine activity *in vivo* of stem cells for tissue engineering applications (Dash et al., 2018) was not effective in counteracting the strong inflammatory response induced by the PP mesh. This hypothesis can be confirmed by the fact that even our histological evaluation did not present significant differences in the infiltration of mononuclear/polymorphonuclear leukocytes in mesh that surrounded tissues at any time point.

The surgical implantation of non-absorbable meshes is associated with a foreign body reaction that leads to fibrous encapsulation of the implant. In the initial host response, proteins and platelets favor the recruitment and adhesion of macrophages and neutrophils; these are followed by lymphocyte infiltration (Klopfleisch and Jung, 2017). We performed a phenotypic characterization of the different leukocyte subsets that infiltrated the surgical mesh and determined the activation status of T helper cells, T-cytotoxic cells, and macrophages. This analysis (performed 1 week and 1 month after mesh implantation surgery) did not reveal any significant differences in the T-cell subsets. We were expecting a macrophage polarization toward M2 cells owing to the immunomodulatory effect of BM-MSCs, according to our previous studies in murine models (Blázquez et al., 2016; Blázquez et al., 2018). The increase in the percentage of tissue-infiltrated CD14^+^ CD163^+^ (M2 cells) in the cell group 1 week after implantation with reference to the control group (**Table 4**), together with the decrease in the expression of the *NOS2* gene (a M1 marker) in the cell group 1 month after implantation, may suggest an M2 polarization by MSCs. However, these changes were not statistically significant. The macrophage analysis also demonstrated a significant decrease in SLA-II expression (SLA-II geometric mean on CD14^+^) in the cell group 1 month after implantation with reference to the control group; nevertheless, the biological significance of this decrease remains uncertain. Hence it is difficult to assert that MSCs triggered an M2 differentiation under these experimental conditions.

The last aim of our study was to evaluate the effects of mesh implantation on connective tissue and vascularization, with or without stem cells. Connective tissue is known to be altered in hernia patients (Henriksen et al., 2011), who thereby present with a low collagen 1/collagen 3 ratio, poor quality collagen, and increased collagen breakdown (Henriksen et al., 2011; Calaluce et al., 2013; HerniaSurge Group, 2018). Moreover, collagen metabolism is strictly related to matrix metalloproteinase (MMP) proteolytic activity in healthy individuals; however, this balance is altered in hernia patients (Henriksen et al., 2011). Stem cells have already been demonstrated to trigger connective tissue remodeling throughout the induction of collagen synthesis and reorganization (Ku et al., 2006; Casado et al., 2014; Liu et al., 2017) and MMP release (Ding et al., 2009; Clarke et al., 2015). For this reason, we performed gene expression analysis of collagens, MMPs, and tissue inhibitors of metalloproteinases (TIMPs) by qPCR and evaluated the histology of connective tissue between and below the mesh areas. However, we found no significant statistical differences in the gene expression of either collagens, MMPs, and TIMPs or their ratios and nor in the histological analysis. Regarding vascularization and angiogenesis, we did not observe any significant changes in vascularization. However, we found a slight, but significant, reduction in *VEGF* expression in the cell group 1 month after mesh implantation. It is true that stem cells have been associated with enhanced angiogenesis in wounds through the release and induction of VEGF (King et al., 2014); however, a high level of angiogenesis has been associated with hypertrophic scarring and fibrosis (DiPietro, 2016), especially in the long term (Karvinen et al., 2011). We hypothesized that stem cells, 1 month after implantation, contributed to the slight reduction in *VEGF*, which thereby minimized severe scarring of the wound.

Altogether, our histological, phenotypic, and gene expression analyses did not reveal any important contribution of stem cell therapy to the implantation of surgical meshes. Nevertheless, this study has established that there remains a lack of knowledge about how to correctly repair hernias with surgical meshes that would guarantee the safety of patients and pose a small risk of adverse effects for them. We recognize that this study has some important limitations. First, our insight led us to rely on a large animal model rather than on small animal models such as rodents, as the large animal model we used is more similar to humans in terms of metabolic requirements, anatomical size, and skin histology. Small animal models with artificially induced abdominal wall defects guarantee the standardization of experimental practices (in this case, similar body mass, sex, and hernia size) and fewer ethical concerns. However, they remain far from clinical practice. Our animal model, in contrast, did not allow the use of large sample sizes and homogeneity: this led to poor significant results in the histological, phenotypic, and gene expression analyses and it is the most important limitation of our study. Second, excluding all the related advantages, laparoscopy has a long learning curve (Hopper et al., 2007) and even expert surgeons need time to practice and standardize this innovative type of surgery. Third, some tests, such as biodistribution or teratogenicity tests, should have been performed to guarantee the safety of the stem cell therapy. We believe that stem cells are not meritless, especially when combined with surgical meshes for hernia repair, but a much larger number of animals, more standardization, and further analyses are required to guarantee reliable results.

To our knowledge, this is the first preclinical study evaluating the use of stem cell therapy in the field of abdominal hernias in a clinically relevant swine model with congenital hernia. According to our study, pigs with congenital hernia closely resemble hernia patients and can be used for further preclinical studies. However, a large number of animals, with similar body masses and hernia sizes, are required to provide consistent results; fibrin sealants can be used to allow cell adhesion on the surgical mesh surface. Moreover, laparoscopy can be used for hernia repair by suturing and it allows for the implantation of surgical meshes seeded with cells. The combined use of meshes and MSCs may allow the creation of bioinert products intended for future clinical applications. This product might have an immediate economic impact by reducing the recurrence of the aforementioned pathologies, hospitalization, and casualties; this product might also have an important impact in the quality of life of patients with hernias. To achieve these aims, extensive and standardized preclinical studies assessing safety and feasibility must be established with urgency.

## Data Availability Statement

The datasets presented in this study can be found in online repositories. The names of the repository/repositories and accession number(s) can be found below: https://figshare.com/, https://doi.org/10.6084/m9.figshare.12287288.v2.

## Ethics Statement

The animal study was reviewed and approved by Ethics Committee on Animal Experiments of the Jesús Usón Minimally Invasive Surgery Centre, in compliance with the recommendations outlined by the local government (Junta de Extremadura), and the EU Directive 2010/63/EU of the European Parliament on the protection of animals used for scientific purposes. 

## Author Contributions

FM, JC, RB, and FS-M conceived and designed the experiments. FD performed the ultrasonography. MB and FS-M performed all the surgical procedures. FM, JC, RB, VÁ, and EL isolated and characterized the cells, prepared the meshes for surgical procedures, and performed molecular and phenotypic analyses. AU prepared histological samples. MS and RM performed the histological evaluations. FM, JC, and RB analyzed the data. FM and JC wrote the article. All authors contributed to the article and approved the submitted version.

## Funding

JUMISC is supported by CIBERCV (CB16/11/00494) and a grant from Junta de Extremadura, Consejería de Economía, Ciencia y Agenda Digital: Ayuda a Grupos Catalogados de la Junta de Extremadura (GR18199) co-financed by European Regional Development Fund (ERDF). This study was also supported by competitive grants, such as: “Miguel Servet I” grant (CP17/00021 and MS17/00021) and project PI18/0911 from Instituto de Salud Carlos III to JC (co-financed by ERDF/ESF); MAFRESA S.L. (Grupo Jorge) grant (promoted by Jesús Usón Gargallo) to FM; Consejería de Economía e Infraestructuras – Junta de Extremadura grant to JC (IB16168 co-financed by ERDF/ESF “Investing in your future”); Sara Borrell grant from Instituto de Salud Carlos III to EL (CD19/00048); CNPqBrazil fellowship (305876/2018-0) to MB. Surgical procedures and imaging diagnoses were performed at the ICTS Nanbiosis (Unit 21, Operating rooms). The funders had no role in study designs, data collection and analysis, decision to publish, or preparation of the article.

## Conflict of Interest

The authors declare that the research was conducted in the absence of any commercial or financial relationships that could be construed as a potential conflict of interest.

## Footnotes

1
https://stemcellsportal.com/stem-cells-translational-medicine-clinical-trials-portal

2
https://baxterpi.com/pi-pdf/Tisseel_PI.pdf

3
https://www.fda.gov/medical-devices/implants-and-prosthetics/urogynecologic-surgical-mesh-implants

4
https://www.fda.gov/medical-devices/implants-and-prosthetics/hernia-surgical-mesh-implants
