# Supplementary material for: Laparoscopy for the Treatment of Congenital Hernia: Use of Surgical Meshes and Mesenchymal Stem Cells in a Clinically Relevant Animal Model
Source: Front Pharmacol. 2020 Sep 25;11:01332. doi: 10.3389/fphar.2020.01332 (PMC7546355; doi:10.3389/fphar.2020.01332)
Supplement: Supplementary file 1 [file DataSheet_1.docx]

Laparoscopy for the treatment of congenital hernia: use of surgical meshes and mesenchymal stem cells in a clinically relevant animal model

Federica Marinaro, Javier G Casado, Rebeca Blázquez, Mauricio Veloso Brun, Ricardo Marcos, Marta Santos, Francisco Javier Duque, Esther López, Verónica Álvarez, Alejandra Usón, Francisco Miguel Sánchez-Margallo

DOI: 10.3389/fphar.2020.01332

Supplementary Material

# Supplementary Figures


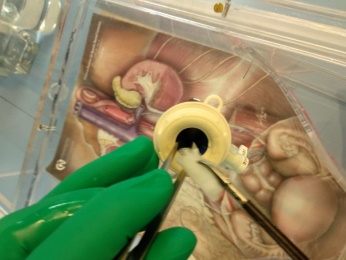

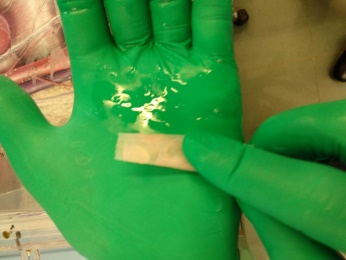

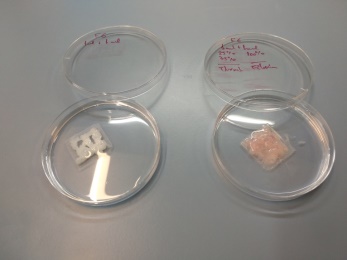

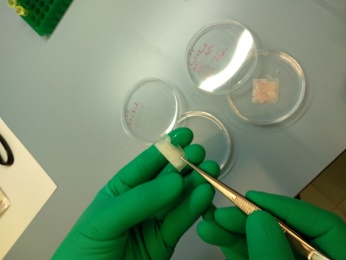


**Supplementary Figure 1**. **Surgical mesh preparation for laparoscopic implantation.** Monofilament polypropylene meshes (Assumesh®, Assut Europe, Italy) were cut with a dimension of 6 x 6 cm. A mixture of cell culture medium and the two components of the fibrin sealant Tisseel® (Baxter) (with or without MSCs) was spread on the top of the surgical mesh with a Tisseel® applicator. The so prepared meshes were rolled and inserted within the trocar for the laparoscopic implantation.

# Supplementary Tables

| **Gene ID** | **Gene name** | **Life Technologies Assay ID** |
| --- | --- | --- |
| *ARG1* | Arginase 1 | Ss03391394_m1 |
| *ACTA2* | Actin Alpha 2, Smooth Muscle | Ss04245588_m1 |
| *BPI* | Bactericidal Permeability Increasing Protein | Ss04321426_m1 |
| *CCL2* | C-C Motif Chemokine Ligand 2 | Ss03394377_m1 |
| *CELA1* | Chymotrypsin Like Elastase 1 | Ss03392393_m1 |
| *COL1A1* | Collagen Type I Alpha 1 Chain | Ss03373340_m1 |
| *COL3A1* | Collagen Type III Alpha 1 Chain | Ss04323794_m1 |
| *CXCL2* | C-X-C Motif Chemokine Ligand 2 | Ss03378360_u1 |
| CXCL8 | C-X-C Motif Chemokine Ligand 8 | Ss03392437_m1 |
| *DES* | Desmin | Ss03378045_u1 |
| *FGF1* (*AFGF*) | Fibroblast Growth Factor 1 | Ss03374827_m1 |
| *FOXP3* | Forkhead Box P3 | Ss03376695_u1 |
| *HPRT1* | Hypoxanthine Phosphoribosyltransferase 1 | Ss03388274_m1 |
| *IFNG* | Interferon Gamma | Ss03391054_m1 |
| *IL1B* | Interleukin 1 Beta | Ss03393804_m1 |
| *IL2* | Interleukin 2 | Ss03392428_m1 |
| *IL4* | Interleukin 4 | Ss03394125_m1 |
| *IL5* | Interleukin 5 | Ss03394369_m1 |
| *IL6* | Interleukin 6 | Ss03384604_u1 |
| *IL10* | Interleukin 10 | Ss03382372_u1 |
| *IL12A* | Interleukin 12A | Ss03391176_m1 |
| *IL13* | Interleukin 13 | Ss03392353_m1 |
| *IL17A* | Interleukin 17A | Ss03391803_m1 |
| *MMP2* | Matrix Metallopeptidase 2 | Ss03394318_m1 |
| *MMP9* | Matrix Metallopeptidase 9 | Ss03392100_m1 |
| *MRC1* | Mannose Receptor C-Type 1 | Ss03373693_m1 |
| *NOS2* | Nitric Oxide Synthase 2 | Ss03374608_u1 |
| *TGFB1* | Transforming Growth Factor Beta 1 | Ss04955543_m1 |
| *TIMP1* | TIMP Metallopeptidase Inhibitor 1 | Ss03381944_u1 |
| *TIMP2* | TIMP Metallopeptidase Inhibitor 2 | Ss03375440_u1 |
| *TNF* | Tumor Necrosis Factor | Ss03391318_g1 |
| *VEGFA* | Vascular Endothelial Growth Factor A | Ss03393993_m1 |

**Supplementary Table 1. Gene ID and commercial references for gene expression assays.** Primers for quantitative polymerase chain reaction were purchased as TaqMan™ gene expression assays from Applied Biosystems (Thermo Fisher Scientific Inc., Waltham, MA, USA). Alternative symbols of genes are reported in brackets.
